# Microwave-Assisted Synthesis and Crystal Structure of Oxo(diperoxo)(4,4'-di-*tert*-butyl-2,2'-bipyridine)-molybdenum(VI)

**DOI:** 10.3390/molecules14093610

**Published:** 2009-09-16

**Authors:** Tatiana R. Amarante, Filipe A. Almeida Paz, Sandra Gago, Isabel S. Gonçalves, Martyn Pillinger, Alírio E. Rodrigues, Marta Abrantes

**Affiliations:** 1 Department of Chemistry, CICECO, University of Aveiro, 3810–193 Aveiro, Portugal; 2 Laboratory of Separation and Reaction Engineering, Associate Laboratory LSRE/LCM, Faculdade de Engenharia da Universidade do Porto, Rua Dr. Roberto Frias, 4200–465, Porto, Portugal

**Keywords:** molybdenum, peroxo complexes, N ligands, microwave-assisted synthesis, X-ray diffraction

## Abstract

The oxodiperoxo complex MoO(O_2_)_2_(tbbpy) (tbbpy = 4,4'-di-*tert*-butyl-2,2'-bipyridine) was isolated from the reaction of MoO_2_Cl_2_(tbbpy) in water under microwave-assisted heating at 120 °C for 4 h. The structure of the oxodiperoxo complex wasdetermined by single crystal X-ray diffraction. The Mo^VI^ centre is seven-coordinated with a geometry which strongly resembles a highly distorted bipyramid. Individual MoO(O_2_)_2_(tbbpy) complexes are interdigitated along the [010] direction to form a column. The crystal structure is formed by the close packing of the columnar-stacked complexes. Interactions between neighbouring columns are essentially of van der Waals type mediated by the need to effectively fill the available space.

## 1. Introduction

Peroxo complexes of high-valent transition metals are used as catalysts or stoichiometric reagents for the oxidation of organic and inorganic substrates [1,2,3,4,5,6,7]. In particular, oxodiperoxo molybdenum complexes bearing bidentate ligands, MoO(O_2_)_2_(L-L), are known to be extremely useful catalysts in the epoxidation of olefins [7,8,9]. A variety of *N*,*N*-chelating ligands have been studied, including 2,2'-bipyridine (bpy) [10], sulfonated bipyridines [11,12], substituted pyrazolylpyridines [8,9,13,14,15,16], oxazolinylpyridines [17], and benzimidazolylpyridines [18]. The standard preparation of these complexes involves the treatment of the organic ligand with MoO_3_ dissolved in H_2_O_2_ (30%).

Some of us recently became interested in the microwave-assisted synthesis (MAS) of metal-organic compounds [19]. Early studies on the use of MAS for metal carbonyl complexes of the type [M(CO)_4_(L)_n_] (M = Cr, Mo, W) indicated that the reaction times could be reduced and the yields increased when compared to conventional methods which use, for example, oil bath heating [20,21,22]. Despite these promising results, the potential use of microwave-accelerated syntheses of metal-organic compounds has hardly been explored, especially when compared with work in organic synthesis using this method [23]. During studies aimed at using MoO_2_Cl_2_(L-L) complexes as precursors to polymeric inorganic/organic hybrid materials, we unexpectedly isolated the oxodiperoxo complex MoO(O_2_)_2_(tbbpy) (tbbpy = 4,4'-di-*tert*-butyl-2,2'-bipyridine) as a by-product of the reaction of MoO_2_Cl_2_(tbbpy) with water under microwave-assisted heating. A detailed description of the synthesis of this compound and its crystal structure are given in this paper. To the best of our knowledge, this is the first report describing the isolation of this type of complex in the absence of an oxidant such as H_2_O_2_ or *tert*-butyl hydroperoxide, that is, using only air as the oxygen source.

## 2. Results and Discussion

### 2.1. Synthesis and spectroscopic characterisation

Treatment of MoO_2_Cl_2_(tbbpy) with water in a microwave oven (dynamic power, 120 °C, 4 h) gave a suspension. After removal of the white solid by filtration in air, acetone was added to the filtrate, and yellow needles of the complex MoO(O_2_)_2_(tbbpy) (**1**) were obtained during slow diffusion of diethyl ether vapour into the water-acetone solution. When the same reaction was carried out under conventional reflux by using oil bath heating instead of microwave-assisted heating, no oxodiperoxo complex was formed. The only product obtained was the white solid, which, on the basis of preliminary analyses, is a polymeric inorganic/organic hybrid compound. Full characterization of this material is still ongoing in our laboratories.

The complex was characterised by FT-IR and Raman spectroscopy, and ^1^H NMR. The FT-IR spectrum of **1** shows two characteristic strong absorption bands at 939 (Mo = O) cm^−1^and 860 (O-O) cm^−1^, a weak shoulder at ca. 870 (O-O) cm^−1^, and a medium intensity band at 1,614 (C = N) cm^−1^. The latter band is indicative of the bidentate coordination mode of the tbbpy ligand to the Mo^VI^ centre [24]. For comparison, the Raman spectrum shows strong bands at 874 cm^−1^, 939 cm^−1^ and 1,610 cm^−1^. The *ν*(M(O_2_)_2_) modes for **1** are located between 500 cm^−1^ and 660 cm^−1^, as found for related complexes [9,15,25]. The Raman spectrum shows one strong absorption at 533 cm^−1^, a medium one at 589 cm^−1^, and a weaker band at 658 cm^−1^. The corresponding bands appear with strong intensity at 530 cm^−1^, 588 cm^−1^ and 657 cm^−1^ in the FT-IR spectrum. The ^1^H-NMR spectrum of **1** shows a set of signals corresponding to the tbbpy moiety at 9.13 (H^6,6'^) ppm, 8.64 (H^3,3'^) ppm, 7.81 (H^5,5'^) ppm and 1.44 (CH_3_) ppm. When compared to the spectrum of the free ligand (doublets at 8.61 ppm and 8.40 ppm, doublet of doublet at 7.49 ppm, and singlet at 1.35 ppm), the set of signals for **1** is shifted to lower field and the resonances are less well defined (such that the H^3,3'^ and H^5,5'^ signals appear as a singlet and a doublet, respectively, i.e., further splitting of the signals due to coupling between the two hydrogens on a given pyridyl ring is not resolved). Taking into account the molecular structure described below, the apparent broadening of the lines in the ^1^H-NMR spectrum of **1** is probably related with the fact that the two peroxo groups and the oxo group are, respectively, directed towards the two different rings, making the protons of each ring slightly non-equivalent.

### 2.2. X-ray crystal structure

**Figure 1:**
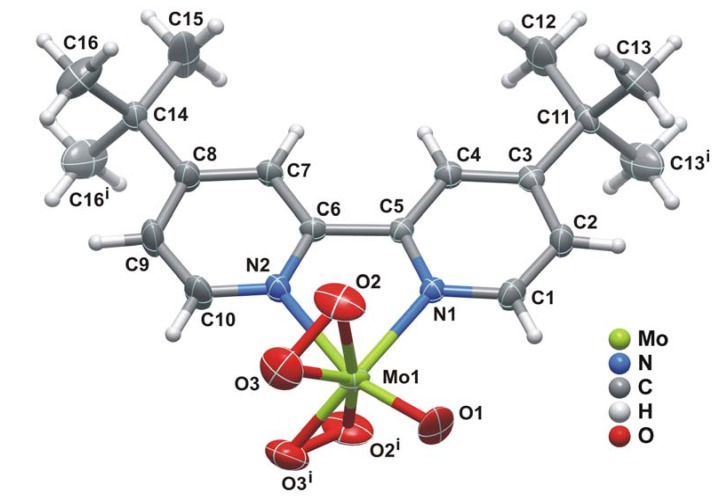
Schematic representation of the neutral MoO(O_2_)_2_(tbbpy) molecular unit present in the crystal structure of **1**, showing the labelling scheme for all non-hydrogen atoms. Thermal ellipsoids are drawn at the 50% probability level and hydrogen atoms are represented as small spheres with arbitrary radii. The disorder associated with the coordinated peroxo (O2'-O3') and oxo (= O1') groups [21.9(7)% occupancy] has been omitted for clarity. Table 1 collects selected bond lengths and angles for the distorted bipyramidal {MoN_2_O_5_} coordination environment. Symmetry transformation used to generate equivalent atoms: (i) *x*, *1.5-y*, *z*.

The structure of **1** (Figure 1) consists of a discrete neutral MoO(O_2_)_2_(tbbpy) complex in which the organic ligand is *N*,*N*-chelated to the Mo^VI^ centre. A search in the literature and in the Cambridge Structural Database (CSD, Version 5.30, November 2008 with 3 updates) for related oxodiperoxo molybdenum complexes revealed only a handful of structures having *N*,*N*-chelated moieties bound to molybdenum centres [10,12,13,14,15,17,26]. A considerable number of these concern derivatives of 4-(1H-pyrazol-4-yl)pyridine such as 3-(2-pyridyl)-1-pyrazolylacetic acid ethyl ester [13], 2-(1-allylpyrazol-3-yl)pyridine [14], (3-(2-pyridyl)-1-pyrazolyl)-cyclohexanol [15] and 2-(1-benzyl-1H-pyrazol-3-yl)pyridine [26]. Only the structures of Schlemper [10] and Herrmann [12] report the use of 2,2'-bipyridine derivatives.

**Table 1:** Selected bond lengths (Å) and angles (°) of the two disordered {MoN_2_O_5_} coordination environments observed for the crystallographically independent MoO(O_2_)_2_(tbbpy) complex in **1**.^a^

Mo1-O1	1.698(5)	Mo1-O3'	1.854(14)
Mo1-O1'	1.757(17)	Mo1-O3	1.876(4)
Mo1-O2	1.939(4)	Mo1-N1	2.235(4)
Mo1-O2'	1.89(5)	Mo1-N2	2.289(4)
O1-Mo1-O2	99.10(18)	O1-Mo1-N1	89.2(2)
O1'-Mo1-O2'	92.3(17)	O2-Mo1-N1	86.20(16)
O1-Mo1-O3	107.89(18)	O1'-Mo1-N1	159.4(6)
O1'-Mo1-O3'	103.8(6)	O2'-Mo1-N1	87.4(18)
O3-Mo1-O2	45.90(19)	O3-Mo1-N1	130.31(14)
O3'-Mo1-O2'	46.4(8)	O3'-Mo1-N1	90.9(5)
O3-Mo1-O2^i^	133.4(2)	O1-Mo1-N2	159.6(2)
O2^i^ -Mo1-O2	160.2(3)	O1'-Mo1-N2	89.0(6)
O2'-Mo1-O2' ^i^	175(3)	O2-Mo1-N2	80.09(17)
O3'-Mo1-O2' ^i^	134.7(12)	O2'-Mo1-N2	88.7(10)
O3'-Mo1-O3' ^i^	88.4(10)	O3-Mo1-N2	86.34(14)
O3^i^ -Mo1-O3	89.1(3)	O3'-Mo1-N2	132.9(5)
O3^i^ -Mo1-O2'	136.7(17)	N1-Mo1-N2	70.39(14)

^a^ Symmetry transformation used to generate equivalent atoms: (i) *x*, *1.5-y*, *z*.

**Figure 2:**
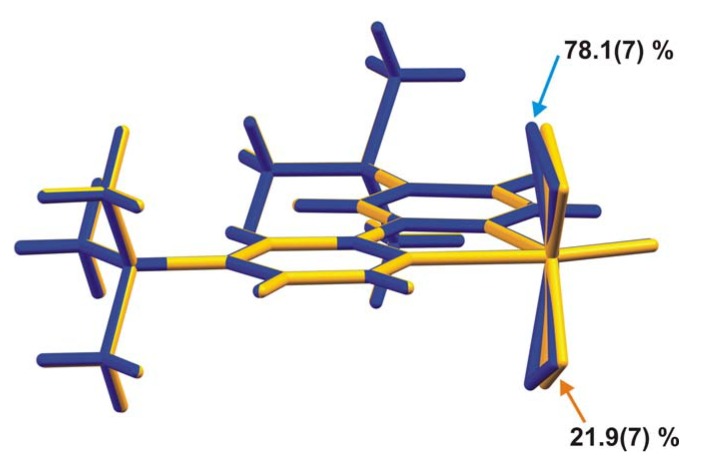
Structure overlay of the two disordered positions for the MoO(O_2_)_2_(tbbpy) complex present in **1**, emphasising the small geometrical differences for the first coordination sphere of Mo^VI^.

**Figure 3:**
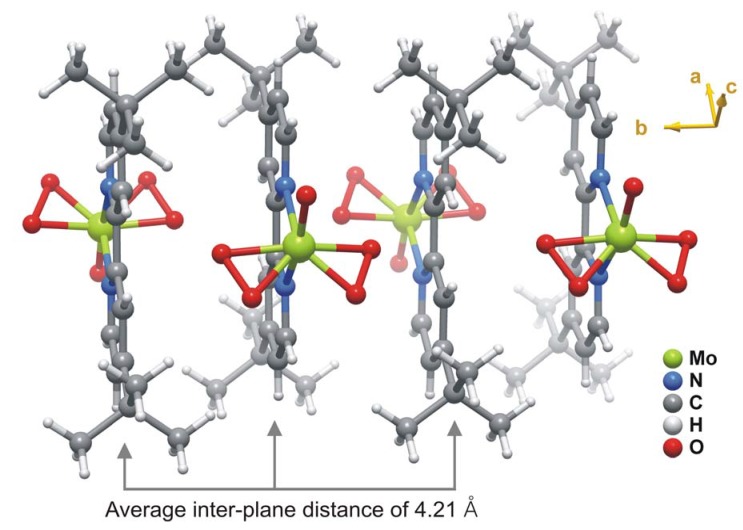
Schematic representation of the interdigitation of individual MoO(O_2_)_2_(tbbpy) complexes along the [010] direction of the unit cell. For simplicity only the most probable locations of the coordinated peroxo and oxo groups are represented.

In the MoO(O_2_)_2_(tbbpy) complex the coordinated tbbpy molecule and the oxo group are structurally located on the mirror plane present in space group *P*nma; the two peroxo groups are crystallographically equivalent and generated by mirror symmetry (Figure 1). These O-containing coordinating groups were found to be structurally disordered over two distinct positions, for which the partial occupancies were modelled to 78.1(7)% and 21.9(7)%. Nevertheless, by rotating the two individual complexes by 180° it is evident that the two complexes are almost identical as shown in Figure 2. Indeed, this disorder in the crystal structure seems to arise from erroneous close packing along the [010] direction: individual MoO(O_2_)_2_(tbbpy) complexes are interdigitated along this direction to form a column as depicted in Figure 3; the ABAB·alternation (i.e., adjacent complexes mutually rotated by 180°) seems to minimise steric repulsion between coordinated groups and, thus, appears as the spatial distribution more frequently observed in the crystal structure. Despite the almost perfect alignment of aromatic rings along this direction, the inter-planar distance of ca.4.21 Å is too long to consider the presence of supramolecular π-π stacking interactions.

The crystallographically independent Mo^VI^ centre is coordinated to two peroxo groups, one oxo group and one *N*,*N*-chelated tbbpy molecule in a seven-coordinated fashion, {MoN_2_O_5_}, with a geometry strongly resembling a highly distorted bipyramid (if one considers the centres of gravity of the peroxo groups as vertices-see Figure 1). The Mo-O_peroxo_ distances lie in the 1.854(14) – 1.939(4) Å range, which agrees well with those reported for related compounds (62 hits in the CSD; 1.80 – 1.99 Å range with median of 1.93 Å).

The steric hindrance directly imposed on the peroxo groups can be inferred from the inter-centroid angle, i.e., we may assume that for close-packed structures the steric pressure on these groups will bring them into closer proximity, resulting in a smaller angle. A search in the CSD reveals that this angle usually varies between 120.2° and 139.9° (median of 129.4°). In **1** the observed value is ca. 135.5°, which is a clear indication that the combination of the columnar arrangement along the [010] direction and the magnitude of the disorder do not imply a considerable steric pressure on the complexes. Taking the centroids of the peroxo groups as the vertices of the coordination polyhedron, it is clear that the resultant bipyramid is considerably distorted: while the equatorial angles vary between ca. 108.0° and 135.5°, those with the apical positions (occupied by the oxo group and one nitrogen atom) are in the 70.4° – 104.6° range.

**Figure 4:**
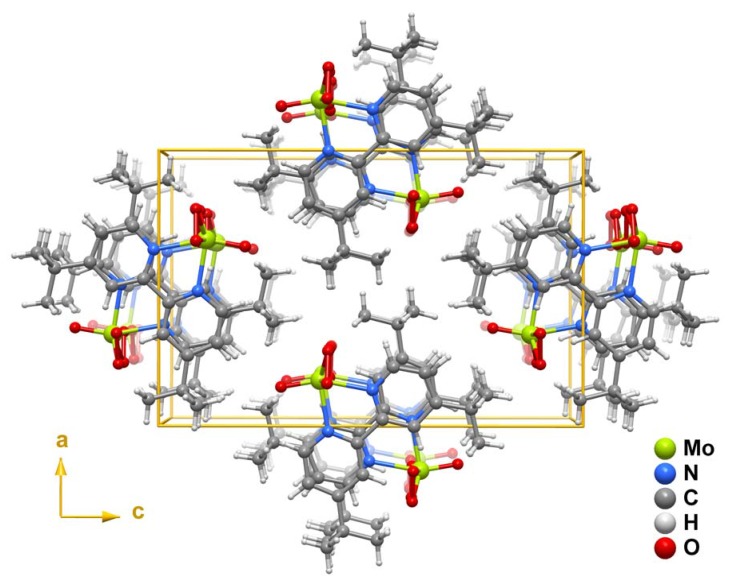
Crystal packing of MoO(O_2_)_2_(tbbpy) (**1**) viewed in perspective along the [010] direction of the unit cell. For simplicity only the most probable locations of the coordinated peroxo and oxo groups are represented.

The crystal structure of **1** (Figure 4) is formed by the close packing of individual columnar-stacked MoO(O_2_)_2_(tbbpy) complexes (as shown in Figure 3). Interactions between neighbouring columns are essentially of van der Waals type mediated by the need to effectively fill the available space.

## 3. Experimental

### 3.1. General

Elemental analysis was performed at the University of Aveiro. FT-IR spectra of undiluted (neat) samples were recorded on a FTIR Mattson-7000 infrared spectrophotometer using a Golden Gate single reflection diamond ATR system with KRS-5 lenses (Specac). Raman spectra were recorded on a Bruker RFS100/S FT instrument (Nd:YAG laser, 1064 nm excitation, InGaAs detector). ^1^H-NMR spectra were measured in solution using a Bruker CXP 300 spectrometer. Chemical shifts are quoted in parts per million from tetramethylsilane. The microwave-assisted synthesis was carried out in a Discover S-Class (CEM Corporation, USA) microwave oven using a glass vessel with a capacity of 35 mL. A dynamic method was used, in which the power was automatically controlled based on the temperature feedback measured using a vertical focused IR sensor. MoO_2_Cl_2_(tbbpy) was prepared according to published procedures [27].

### 3.2. MoO(O_2_)_2_(tbbpy) *(**1**)*

A mixture of MoO_2_Cl_2_(tbbpy) (1.00 g, 2.15 mmol) and water (25 mL) was stirred and heated to 120 °C inside the microwave oven and maintained at this temperature for 4 h. At the end of the reaction a white precipitate and a pink solution (pH ≈ 2) were obtained. The solid was removed by filtration in air, and acetone (10 mL) was added to the solution. Slow diffusion of diethyl ether vapour into the water-acetone solution over a period of two months afforded yellow crystals of **1** (114 mg, 12%). Anal. Calcd for C_18_H_24_MoN_2_O_5_ (444.33): C, 48.66; H, 5.44; N, 6.30. Found: C, 48.76; H, 5.69; N, 5.93%; FT-IR (cm^−1^): *ν* = 2,967m, 2,873w, 1,614s, 1,550m, 1,484m, 1,461w, 1,411s, 1,373m, 1,305w, 1,251s, 1,205w, 1,124w, 1,081w, 1,025m, 939vs, 900m, 870sh, 860vs, 846sh, 744m, 719m, 657s, 603w, 588s, 553m, 530s, 430m, 401m, 358m, 327m, 308w, 304w; Raman (cm^−1^): *ν* = 3,086s, 2,971vs, 2,936w, 2,909vs, 2,876w, 1,610s, 1,543s, 1,491m, 1,448w, 1,418s, 1,371m, 1,321vs, 1,254m, 1,205m, 1,128m, 1,033vs, 939vs, 918m, 902w, 874s, 804m, 722s, 658w, 589m, 554w, 533s, 405w, 297m, 248m, 172m, 126s; ^1^H-NMR (300.13 MHz, 25 °C, dimethylsulfoxide-d_6_): *δ* = 9.13 (d, 2H, H^6^ and H^6'^, *J*(H-H) = 6 Hz), 8.64 (s, 2H, H^3^ and H^3'^), 7.81 (d, 2H, H^5^ and H^5'^, *J*(H-H) = 6 Hz), 1.44 (s, 18H, CH_3_) ppm.

### 3.3. Single crystal X-ray diffraction

Single-crystals of MoO(O_2_)_2_(tbbpy) (**1**) were quickly transferred from the mother liquor to a glass vial containing highly viscous FOMBLIN Y perfluoropolyether vacuum oil (LVAC 140/13) purchased from Sigma-Aldrich. A suitable crystal was mounted on a Hampton Research CryoLoop [28] with the help of a Stemi 2000 stereomicroscope equipped with Carl Zeiss lenses. X-ray data were collected on a Bruker X8 Kappa APEX II CCD area-detector diffractometer (Mo K_α_ graphite-monochromated radiation, *λ* = 0.71073 Å) controlled by the APEX2 software package [29], and equipped with an Oxford Cryosystems Series 700 cryostream monitored remotely using the software interface Cryopad [30]. Images were processed using the software package SAINT+ [31], and data were corrected for absorption by the multi-scan semi-empirical method implemented in SADABS [32]. The structure was solved using the Patterson synthesis algorithm implemented in SHELXS-97 [33,34], which allowed the immediate location of the metal centre. All remaining non-hydrogen atoms were located from difference Fourier maps calculated from successive full-matrix least squares refinement cycles on *F*^2^ using SHELXL-97 [33,35].

The oxo and peroxo groups coordinated to the metal centre were found to be structurally disordered over two distinct positions. This disorder was modelled into the final structure by considering refineable occupancy rates for each set of coordinated groups [O1 and O2 = O3; O1' and O2' = O3'] which ultimately converged to 78.1(7) and 21.9(7)%, respectively. Figure 1, Figure 3, Figure 4 show only the most probable location for these atoms. Nevertheless, geometrical parameters of the distorted bipyramidal {MoN_2_O_5_} coordination environment concerning the two crystallographic locations for these coordinated groups are given in Table 1. With the exception of O2', all non-hydrogen atoms were successfully refined using anisotropic displacement parameters.

Hydrogen atoms bound to carbon were located at their idealised positions using appropriate *HFIX* instructions in SHELXL (*43* for the aromatic and *33* for the terminal -CH_3_ methyl groups) and included in subsequent refinement cycles in riding-motion approximation with isotropic thermal displacements parameters (*U*_iso_) fixed, respectively, at 1.2 or 1.5 times *U_eq_* of the carbon atom to which they are attached. The last difference Fourier map synthesis showed the highest peak (0.984 eÅ^−3^) and deepest hole (−1.715 eÅ^−3^) located at 1.33 Å and 1.56 Å from H12A and H16B, respectively.

Information concerning crystallographic data collection and structure refinement details is summarised in Table 2.

**Table 2:** Crystal and structure refinement data for MoO(O_2_)_2_(tbbpy) (**1**).

Formula	C_18_H_24_MoN_2_O_5_
Formula weight	444.33
Crystal system	Orthorhombic
Space group	*P*nma
*a*/Å	12.0720(5)
*b*/Å	8.4233(3)
*c*/Å	18.5275(7)
Volume/Å^3^	1883.99(13)
*Z*	4
*D*_c_/g cm^−3^	1.567
*μ*(Mo-Kα)/mm^−1^	0.727
F(000)	912
Crystal size/mm	0.16 × 0.06 × 0.05
Crystal type	Yellow needles
*θ* range	3.55 to 29.13
Index ranges	−16 ≤ *h*≤ 11, −11 ≤ *k*≤10, −25 ≤ *l* ≤ 25
Data completeness to *θ* = 29.13	98.9%
Reflections collected	24626
Independent reflections	2676 (*R*_int_ = 0.0351)
Final *R* indices [I > 2σ(I)]^a,b^	*R*1 = 0.0470, *wR*2 = 0.1009
Final *R* indices (all data)^a,b^	*R*1 = 0.0612, *wR*2 = 0.1050
Weighting scheme^c^	*m* = 0.0200, *n* = 5.4626
Largest diff. peak and hole	0.984 and −1.715 eÅ^−3^

^a^ 
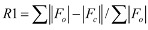
; ^b^ 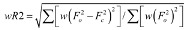
^c^ 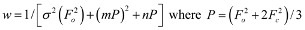

CCDC 738526 contains the supplementary crystallographic data for this paper. These data can be obtained free of charge via www.ccdc.cam.ac.uk/conts/retrieving.html (or from the CCDC, 12 Union Road, Cambridge CB2 1EZ, UK; Fax: +44 1223 336033; Email: deposit@ccdc.cam.ac.uk)

## 4. Conclusions

The isolation of the oxodiperoxo complex MoO(O_2_)_2_(tbbpy) in significant amounts from the reaction of MoO_2_Cl_2_(tbbpy) with water under air is surprising, since all known standard procedures for the synthesis of this type of complex involve a peroxide source such as H_2_O_2_ or *tert*-butyl hydroperoxide (TBHP). For example, we recently found that complex **1** is formed upon treatment of the thiocyanate complex MoO_2_(NCS)_2_(tbbpy) with an excess of TBHP [25]. The use of microwave-assisted heating seems to play a role in the formation of complex **1** from MoO_2_Cl_2_(tbbpy) and water, although further investigation is required to fully delineate its influence compared with conventional heating methods.
